# The past, present and future of breeding rust resistant wheat

**DOI:** 10.3389/fpls.2014.00641

**Published:** 2014-11-24

**Authors:** Jeffrey G. Ellis, Evans S. Lagudah, Wolfgang Spielmeyer, Peter N. Dodds

**Affiliations:** Commonwealth Scientific and Industrial Research Organisation, Agriculture FlagshipCanberra, ACT, Australia

**Keywords:** wheat rust, *Puccinia*, disease resistance gene, adult plant resistance (APR), wheat biotechnology, GM wheat

## Abstract

Two classes of genes are used for breeding rust resistant wheat. The first class, called R (for resistance) genes, are pathogen race specific in their action, effective at all plant growth stages and probably mostly encode immune receptors of the nucleotide binding leucine rich repeat (NB-LRR) class. The second class is called adult plant resistance genes (APR) because resistance is usually functional only in adult plants, and, in contrast to most R genes, the levels of resistance conferred by single APR genes are only partial and allow considerable disease development. Some but not all APR genes provide resistance to all isolates of a rust pathogen species and a subclass of these provides resistance to several fungal pathogen species. Initial indications are that APR genes encode a more heterogeneous range of proteins than R proteins. Two APR genes, *Lr34* and *Yr36*, have been cloned from wheat and their products are an ABC transporter and a protein kinase, respectively. *Lr34* and *Sr2* have provided long lasting and widely used (durable) partial resistance and are mainly used in conjunction with other R and APR genes to obtain adequate rust resistance. We caution that some APR genes indeed include race specific, weak R genes which may be of the NB-LRR class. A research priority to better inform rust resistance breeding is to characterize further APR genes in wheat and to understand how they function and how they interact when multiple APR and R genes are stacked in a single genotype by conventional and GM breeding. An important message is do not be complacent about the general durability of all APR genes.

## INTRODUCTION

Three wheat diseases, stem, leaf, and stripe (or yellow) rust, caused by *Puccinia graminis* f. sp. *tritici* (Pgt), *P. triticina* (Ptr), and *P. striiformis* f. sp *tritici* (Pst), respectively, cause important losses of grain production (McIntosh et al., 1995). Pgt, and in particular the broadly virulent African strain Ug99, is on many wheat breeders’ minds because this disease had been considered generally under control. There are two ways to control rust in cereals, chemical control and genetic resistance. Genetic control has advantages for environmental and economic reasons, particularly for farmers in the developing world, and because of the possibility that rust pathogens develop resistance to fungicides (Oliver, 2014). When it comes to genetic resistance used by wheat breeders there are two general classes of genes based on their phenotypic effects, pathogen race- or strain-specific resistance (R genes) and adult plant resistance (APR) genes. R genes mostly function from seedling to adult growth stages whereas APR genes function mainly at the adult stage. Wheat rust resistance genes of both R and APR classes are designated *Lr*, *Sr,* and *Yr* (for leaf, stem, and stripe or yellow rust resistance, respectively) without distinction between R or APR classes and with increasing numbers to accommodate newly discovered genes.

Currently there is a view among some breeders and pathologists that more emphasis should be placed on discovery, characterization and use of APR genes for durable resistance (i.e., long lasting when broadly deployed in agriculture) with an implicit suggestion that less emphasis be given to using resistance (R) genes because their lack of durability. From the outset, we state our position is that when it comes to combating rust, use every genetic tool available. In this review we look at the present state of knowledge of wheat rust resistance genes and application in resistance breeding. We revisit some of the history of the area to refine current thinking in terms of new and historical research findings and consider the future use of R and APR genes in wheat breeding. Although the focus will be on rusts, other recent advances in disease resistance studies will be incorporated when instructive.

## USING R GENES FOR RUST RESISTANCE BREEDING

Resistance genes are the better defined of the two classes of resistance genes in terms of classical and molecular genetics and extensively used class in breeding programs. This class is also referred to as *‘major gene resistance,’ ‘gene-for-gene resistance’ ‘race specific resistance,’* and *‘seedling resistance*,’ although with very few exceptions, resistance extends from seedling stage into adult stages, the major growth periods for rust damage. The genes in this class mostly conform to Flor’s gene-for-gene hypothesis (Flor, 1971). Two key genes are necessary for expression of resistance; the R gene in the host and the corresponding avirulence (Avr) effector gene in the rust pathogen. Each R gene confers resistance to pathogen strains carrying the corresponding Avr effector gene. In other words, the efficacy of R genes is pathogen strain dependent. The ability of the pathogen to overcome resistance derives from mutation of the Avr gene leading to loss of recognition by the corresponding R gene. R genes have been isolated from many plant species including wheat (see below), and encode receptor proteins that either directly or indirectly recognize pathogen Avr proteins. Many Avr genes have been isolated from various plant pathogens and they typically encode proteins that are secreted into the host to promote infection. This area of disease resistance has been extensively reviewed (e.g., Dodds and Rathjen, 2010) and here we discuss only some new information, opinions and concepts pertinent to wheat rust.

Some R genes in crops have been referred to as “broad spectrum” because they confer resistance to all tested races of a single pathogen species. We question whether this term is inappropriate and not misleading for R genes. First it implies that that these genes are somehow different from “narrow spectrum” R genes that confer resistance to only some isolates of the pathogen, which they are not and secondly that they will be more durable, which they will also not be. *The only difference between the former and latter R genes is that the former recognize Avr genes present in all pathogen isolates tested at a particular point in time.* While such genes are desirable for disease control, there is no *a priori* reason to expect this class to have increased durability. The situation of “broad spectrum” R genes often occurs when an R gene is newly introduced into a cultivated crop species from a wild or domesticated relative species (e.g., wild *Solanum* sp into potato, rye into wheat) and the pathogen in the crop situation has not been previously exposed to selection by the so-called “broad spectrum” gene. Once strong selection is applied to these genes, previously avirulent pathogen races are likely to evolve virulence. Examples in wheat are the *Sr24* gene from *Agropyron* sp., which was overcome in many areas quite rapidly whereas in Australia it has remained effective (McIntosh et al., 1995) and *Sr31* from rye, which was effective worldwide against all Pgt races for 30 years until the appearance of Ug99 (Pretorius et al., 2000). Why *Sr31* remained effective in contrast to other Sr genes for this time is an intriguing question for research.

R genes were the first class of resistance genes to be genetically defined, and their very clear phenotypic effects with high levels of resistance conferred by single genes that can be rapidly detected in glasshouse tests using seedling plants made selection simple and economical and hence they were rapidly adopted by wheat breeders. However, soon after these genes began to be used in breeding programs in the early to mid 20th century it became very clear that new virulent strains would arise that overcame single resistance genes in new varieties often within a few years of release. The virulent strains were either present at a low frequency in the existing pathogen populations, or were derived later by sexual re-assortment of existing genetic variation for or mutation toward virulence. In other words, these genes used singly are not durable in agriculture. Nevertheless with good management, R genes have been (and should continue to be) used with considerable success to control stem rust in North America, Australia, and other parts of the world. The longer term success of R gene breeding derives from the use of varieties carrying several genes effective against most and preferably all the local rust races (gene pyramids or stacks) meaning that where pathogen mutation is the source of virulence, rare, multiple, independent mutations in different Avr genes are required for evolution of virulence in the pathogen. Success in relation to stem rust control has also been assisted by the near absence (Australia) or extensive reduction (North America) of the main alternate host *Berberis vulgaris*, which is necessary for sexual recombination and re-assortment of new ‘resistance breaking’ combinations of Avr genes not recognized by the currently deployed R genes (Leonard and Szabo, 2005). Additionally the agronomic practice of planting only resistant varieties and reduction or prevention of inter-seasonal survival of the rust species on volunteer wheat and other susceptible species has a major effect on R gene durability by driving the pathogen population size down with the result of greatly reduced likelihood of virulent strains arising from low frequency events such as mutation. Another important factor is that many national and now global rust control programs monitor pathogen virulence phenotypes and provide information about frequencies of rust pathogen races with virulence to particular R genes and R gene combinations (Singh et al., 2011b). This knowledge can be used by breeders to anticipate and respond to new and dangerous races identified inside and outside of their breeding regions, as has been seen in the international response to the African stem rust race group Ug99. This response can, however, be insufficiently timely or vigorous because, as recognized for many years (Waldron and Clark, 1936), it is difficult to get farmers to abandon good cultivars in favor of newer resistant ones on the basis of warnings of potential future rust pathogenicity changes unless the newer resistant cultivars are actually as good as or better yielding than the existing ones. A weakness with the use of R genes is the difficulty in assuring that the best and most durable combination of R genes (“gene stewardship”) are deployed effectively across international frontiers and more frustratingly, even national regions, across which new rust pathogen races can easily spread by wind.

Intriguingly, compared to stem rust control, R genes have been relatively unsuccessful in controlling stripe rust in many parts of the world. In Australia, Europe, and North America perusal of *Yr* genotypes of common wheat varieties indicates less genetic variation and less frequent complex gene combinations compared to *Sr* genes. In Australia, the introduction of a new race of stripe rust in 2002, which due to its very different Avr/virulence characteristics compared to the first 1979 incursion of Pst, overcame many of the R (and indeed some APR!) genes that were being used effectively by breeders. This sudden race change rapidly reversed previous resistance breeding gains made against the 1979 incursion and its mutational derivatives and even now, 12 years after the 2002 incursion, breeders have not reached a stage where chemical control is not needed, often because single R genes were relied on and evolution toward virulence on these genes was rapid. Perhaps this situation could be due, unlike Centro Internacional de Mejoramiento de Maíz y Trigo (CIMMYT; International Maize and Wheat Improvement Centre) breeding, to Australian rust pathology and wheat breeding no longer being closely coordinated as a joint enterprise.

Many wheat rust resistance genes have been introgressed into wheat from its wild and cultivated relatives (so-called “alien” genes) by interspecific hybridization. A large proportion of these genes are not yet in commercial cultivars because of the presence of large alien chromosome segments carrying negative traits linked to the resistance gene (so-called linkage drag). With the advent of Pgt strains in the Ug99 lineage with virulence toward most of the commonly used R genes present in current commercial varieties (Pretorius et al., 2000), the introgression of alien genes and reduction of the size of the associated alien chromosome segment by cytogenetic methods (“chromosome engineering”), has re-emerged as an important active area contributing re-engineered and new R genes to wheat breeding. Genome information and associated high through put technologies makes major contributions by providing quicker access to accurate linked DNA markers for rapid selection of induced wheat-alien chromosome recombination events that minimize the extent of alien DNA (Niu et al., 2011; Mago et al., 2013). The absence of linkage drag is only determined by the “survival” of the alien-derived R gene during breeders’ selection for yield and quality and then appearance in a new variety. Screening large numbers of accessions of the wild D genome donor of wheat, *Aegilops tauschii* and use of rust resistance in this species also offers a source of novel resistance for wheat and introgression can be achieved more readily via synthetic hexaploids resulting from tetraploid wheat crosses to *Aegilops tauschii* (Rouse et al., 2011). In these hybrids the lack of recombination suppression between the D genomes of domesticated and synthetic wheat greatly assists in getting these new genes into varieties and by passes some of the more complex procedures involved in “chromosome engineering” such as genetic induction of recombination between homoeologous wheat and alien R gene donor chromosomes.

Cloning resistance genes has provided molecular insights into rust resistance. *L6* from flax (linseed) was the first rust resistance gene cloned in Lawrence et al. (1995). *Rp1-d* from corn, was the first cereal rust resistance gene was cloned in Collins et al. (1999). Subsequently wheat leaf rust resistance genes *Lr10*, *Lr1*, *Lr21* (Huang et al., 2003; Cloutier et al., 2007; Loutre et al., 2009) stem rust resistance genes *Rpg1* and *Rpg5* from barley (Brueggeman et al., 2002, 2008), and stem rust resistance genes *Sr33* and *Sr35* (Periyannan et al., 2013; Saintenac et al., 2013) from wheat were cloned. All the cereal rust R genes (except *Rpg1*) belong to the coiled–coil, nucleotide binding site, leucine rich repeat (CC-NB-LRR) class of R proteins. Consequently basic research on resistance gene function in cereals as against model systems is beginning to expand. It is now 20 years since the first cloning of this class and many reviews have covered the progress in understanding of these proteins but some recent developments are discussed (Qi and Innes, 2013).

One important new development has been a rush of discoveries has identified a novel class of NB-LRR genes arranged as closely linked, divergently transcribed pairs (1 to several Kbs of DNA apart) and where resistance requires the function of both genes (Cesari et al., 2014b; Williams et al., 2014; Zhai et al., 2014). Their protein products interact to form dimers or higher order complexes. One member often carries at the *C*-terminus, or otherwise embedded within the protein, one of several unique non-NB-LRR pathogen effector sensing domains. It appears likely that these sensor domains have been acquired through DNA transfers during evolution from other host genes whose products (so-called ‘virulence targets’) and functions are targeted, manipulated and perturbed by pathogen effectors transferred from pathogen to host during infection. The second member of the NB-LRR pair acts as the signal transducer, signaling the presence of the pathogen and activating the still poorly defined downstream host defense machinery. These gene duos are important in rice for blast resistance (Cesari et al., 2014b) Relevant to this review, one pair has been described at the leaf rust resistance locus *Lr10* (Loutre et al., 2009) in wheat and another at the *Rpg5* stem rust resistance locus in barley (Wang et al., 2013). Why R genes like *Sr33* (Periyannan et al., 2013) can act singly while others function as pairs is an intriguing question (Cesari et al., 2014a). Paired proteins with complementary activities may allow R protein function that would otherwise be disrupted when single R proteins fuse with amino acids sequences from virulence targets. Fusion of R proteins to copies of guarded host target or decoy proteins must increase the thermodynamic efficiency of detection of pathogens above that of separate proteins that require diffusion and chance interaction in the cytosol of independent receptor (R) and host target-effector interacting proteins to activate defense.

A notable feature of resistance responses of R genes is localized cell death (hypersensitive response or HR) the extent of which and degree of resulting pathogen inhibition differs between R genes. For wheat stem rust for example, infection is described on a 0–4 scale with the 0 extreme signifying no visible presence of the pathogen (complete resistance) and the four extreme signifying infection sites with large pustules producing copious quantities of infectious urediospores (complete susceptibility). However, many wheat rust resistance genes express intermediate type 2 seedling infection type, which is characterized by small sporulating pustules with green islands surrounded by HR and/or chlorosis, whereas rarely some like *Sr5* give complete immunity. These phenotypes can be dramatically seen by simply flicking through the photographs of different R gene dependent resistance in ‘wheat rusts: an atlas of resistance genes’ (McIntosh et al., 1995, http://www.globalrust.org/sites/default/files/wheat_rust_atlas_full.pdf). We refer to this intermediate level of resistance as “incomplete resistance,” retaining the term “partial resistance” for the APR phenotypes described later. The molecular basis of different levels of R gene resistance was not understood and is being studied in our lab. NB-LRR proteins have are three separate functions: (1) pathogen strain (effector) recognition, (2) R protein transition from the resting to active state and (3) signaling to the host defense response machinery. We propose that R proteins are enzymes poised in equilibrium between the active and inactive state. The output of the active state is dangerous to the plant in the absence of the pathogen because it results in cell death and extreme dwarfism when expressed systemically (Howles et al., 2005). Therefore there needs to be mechanisms to ensure that the equilibrium is shifted toward the inactive state when the pathogen Avr proteins are absent. These mechanisms include intra-molecular interactions in R proteins (Luck et al., 2000; Hwang and Williamson, 2003), inter-molecular R protein – repressor protein interactions (for example RPS2 and RIN4, Axtell and Staskawicz, 2003) and possible siRNA pathways modulating the level of R protein mRNA (Li et al., 2012; Shivaprasad et al., 2012). Selection may act on each of these mechanisms for control of R protein defense signal output.

Work we have done recently with flax rust provides insight into one example of an incomplete resistance gene that may turn out to be a more general but not the only explanation for incomplete rust resistance. The allelic *L6* and *L7* genes in flax are members of the dicot specific Toll Interleukin-1 Receptor class of R genes (the TIR-NB-LRR class, absent from wheat). *L6* and *L7* proteins recognize and interact with AvrL567 Avr proteins but *L6* plants express full resistance (type 0 equivalent in the wheat stem rust system) and *L7* an incomplete resistance (type 2 equivalent) toward avirulent pathogen isolates. DNA sequencing of *L6* and *L7* indicated that they encode proteins that differ by 11 amino acid differences in the TIR domain which was recently shown to be the domain involved in signaling to the host defense machinery (Bernoux et al., 2011). Our recent results indicate that these differences maintain the L7 protein more strongly in the inactive state compared to L6, reducing the overall level of defense activation, which is observed as higher levels of rust infection on *L7* plants compared to *L6*. In simple terms, L7 is more difficult to activate that L6 so the level of resistance is lower. In nature, such a situation, leading to incomplete resistance to a pathogen on one hand (a lower level of resistance) and potentially less ‘leakage’ of defense signal and consequent negative effects on the plant in the absence of the pathogen, is likely to be subject to balancing selection. So the high frequency of incomplete resistance in plants may be a result of compromise between selection for low levels of activation in the absence of pathogen triggers of the many R genes in plant genomes and selection for sufficient activation in the presence of the pathogen to provide disease resistance. A similar phenomenon was recently described for the CC-NB-LRR protein Pm3 for powdery mildew resistance in wheat where two amino acid substitutions in the nucleotide binding domain enhance the resistance phenotype and increase the range of mildew isolates recognized, presumably because the Avr proteins produced by these isolates activate the mutant and not wild type *Pm3* (Stirnweis et al., 2014).

Some breeders perceive R genes as problematic in agriculture because their effects are generally not durable, particularly if deployed singly, as a direct result of the nature of these genes conferring a recognition-based resistance that can be overcome by mutation of the pathogen’s corresponding Avr gene. Many examples of so-called “boom-and-bust” cycles in agriculture can be cited where R genes have been (and continue to be) used injudiciously, resulting in active encouragement by the same breeders and pathologists to move away from R gene use toward exclusively using the APR class of genes discussed later. This raises the consideration of the value of R genes in nature. R genes are abundant in wild plant populations as evidenced even by wheat breeding where genes introgressed into wheat have come from wild relatives. R genes are also abundant in wild *Arabidopsis thaliana* and wild Australian indigenous flax, *Linum marginale*, which contains a great diversity of R specificities against flax rust (Burdon, 1994; Nemri et al., 2010). Maintenance of these genes and their diversity in wild plant populations indicates their importance in natural ecosystems. In general in wild systems *individuals carry at least one resistance gene effective against some but not all isolates of an adapted pathogen species and varying levels of disease occur from year to year* (Burdon, 1994). Consequently each pathogen isolate can grow on some but not all host isolates. So what selective forces maintain this patchwork of diversity of R genes in wild populations given what could be seen as an ineffective (at least incomplete) disease control? There must be sufficient selective advantage to maintain these genes in a host by virtue of being resistant to some proportion of the pathogen population as against being susceptible to all of the pathogen population, resulting in reduced disease load. When pathogen spores land on resistant plants, the ability of this population of spores to contribute to epidemic progression is removed. Maintenance of diversity in gene-for-gene resistance in natural populations argues that R genes are selectively advantageous in reducing pathogen damage that would otherwise have lead to host extinction. Mimicking this situation in agriculture by using “multi-lines,” with each line carrying a different R gene, was a much investigated area in the second half of the 20th century but as far as we are aware, this is not a control measure that is currently used in wheat production (Wolfe, 1988). The presence of some but not complete resistance to a pathogen in natural populations is sufficient for host species (and pathogen) survival but may not be sufficient for disease control and productivity in modern agriculture.

Another way of thinking about the selective value of R genes in nature, which has not been considered in evolutionary models, is partly based on the concept of the equilibrium between active and inactive R protein states that was discussed above. Since resistance proteins are enzymes and it is difficult to switch enzymatic activity to zero, there is likely to be genetic variation that affects the equilibrium level of activity and defense signal output for many of the R proteins encoded in plant genomes. Experimental molecular studies have shown that plants carrying induced high signal activity mutations in NB-LRR genes, which are referred to as autoactive mutants, express defense response genes such as *PR1* and have higher levels of resistance to virulent pathogen strains. For example, flax plants expressing weak autoactive phenotypes due to an *L6* rust resistance gene mutation express incomplete resistance to flax rust strains lacking the corresponding Avr gene *AvrL567* (Howles et al., 2005). Interestingly, when the rust is halted by this general leaky defense, the infection site becomes necrotic (HR) raising the hypothesis that the HR response in plants is a default reaction when pathogen growth (and thus effector production) is inhibited. However, flax plants expressing autoactive R gene mutants show various levels of growth inhibition, ranging from minor to major degrees of dwarfism depending on the degree of defense activation (Howles et al., 2005). Thus, leaky defense signaling from some R genes may provide general resistance against diverse pathogens even in the absence of specific Avr recognition, and this may contribute to selection for R genes even when specifically avirulent pathogens are not present. However, it may also impose a cost to the host in the absence of pathogen pressure.

In natural populations, overall defense signaling output can occur at the level of presence or absence polymorphisms of specific R genes in individuals. For example the *RPM1* gene imposes fitness costs in *Arabidopsis* and a presence/absence polymorphism occurs in wild populations (Tian et al., 2003; Karasov et al., 2014). Polymorphism similar to those observed for the *L6* and *L7* allelic variants in flax may also ocurr, leading to different activation thresholds determined by intramolecular interactions within the immune receptor protein. Polymorphisms may also occur in transacting proteins that result in varying degrees of repression of R protein activity in the absence of pathogens. So in any plant population, this hypothesis predicts that there will be variation between individuals in levels of spontaneous (“leaky”) defense responses in the absence of specific recognition, which could lead to either a fitness cost in the absence of pathogens, or be advantageous under disease pressure.

Consistent with this hypothesis, a recent paper (Karasov et al., 2014) showed that maintenance of RPS5 in wild *Arabidopsis* populations could not be explained by the presence of the corresponding Avr gene (*AvrPphB*) in *Pseudomonas syringae* populations alone and postulated that RPS5 may confer a resistance phenotype against other pathogens. Whether the fitness costs of RPS5 and RPM1 (Tian et al., 2003) are due to constitutive defense activation, and whether this results in a level of non-specific disease resistance has neither been considered nor tested in these papers, for example. by quantifying PR1 defense marker expression in the presence or absence of these genes. Indeed, balancing selection has been observed in natural populations of *Arabidopsis* between polymorphic autoactive forms of the defense protein ACD6 (not an NB-LRR), with plants with low to quite high levels of constitutive defense gene expression favored in locations and years of high pathogen pressure and such phenotypes selected against in years or locations of low pathogen pressure (Todesco et al., 2010, 2014). Constitutive defense activation can also occur in F1 hybrids of certain *Arabidopsis* accessions as a result of interactions between two polymorphic genes usually including at least one NB-LRR protein (Bomblies et al., 2007). Furthermore, there are several examples from *Arabidopsis* in which expression of several R genes are induced by various compatible pathogen infections possibly leading to increased R protein and signal leakage (Navarro et al., 2004). So in any wild plant population, this hypothesis predicts that there will be variation between individuals in the levels of spontaneous resistance response in the absence of pathogens.

Thus in addition to gene-for-gene isolate specific resistance, NB-LRR protein (or regulatory protein) polymorphisms have the potential to provide a general pathogen resistance because of defense signal leakage combined with a fitness cost in the absence of pathogen pressure that could lead to the maintenance of these R gene polymorphisms in natural populations by balancing selection. It may be expected that in modern agriculture where yield is the principal target for selection, these sorts of resistances with an associated yield cost would be eliminated by plant breeding. However, in agriculture breeders also balance the economic advantages of pathogen resistance with minor yield costs, for example, in some environments the widely used APR gene, *Lr34*, has a 9% yield cost in the absence of pathogens in some environments (Singh and Huerta-Espino, 1997). Do these sorts of polymorphisms in R genes and their regulatory pathways account for some of the weak adult plant resistance (APR) phenotypes discussed in the next section?

## ADULT PLANT RESISTANCE (APR) GENES FOR RUST RESISTANCE BREEDING

Adult plant resistance genes express partial rust resistance phenotypes only in adult plants (except under very specific conditions) and this is characterized by less and slower pathogen growth without a necrotic response (sometimes referred to as “slow rusting”). Consequently APR is selected by wheat breeders in the field and not in the glasshouse. Reliable field sites for disease assays that optimize natural infections or induced epidemics for effective selection of resistance must be available. The masking of APR by of R genes with stronger resistance phenotypes can prevent effective APR selection unless specific races of pathogens are used to induce epidemics. All these factors make wheat breeding with APR genes more complex than using R genes. Although the resistance phenotypes conferred by individual APR genes show varying levels of partial resistance, it has been reported that when several, mainly undefined APR genes are combined, “near immunity” can be achieved in adult field grown plants (Singh et al., 2014).

The best known APR genes in wheat are *Sr2,* a stem rust resistance gene and *Lr34*, a gene that provides resistance to leaf and stripe rust and powdery mildew. These genes have been used in commercial wheat varieties for almost 100 years. *Sr2* and *Lr34* have provided partial resistance for many years over large areas and under high and prolonged disease pressure in the field, hence they have proven durable (Johnson, 1984). Importantly neither APR genes on their own provides adequate levels of resistance under high disease pressure and often APR expression can be too late in the field to adequately protect yield. The slow rusting and quantitative nature of their phenotypes have incorrectly led to misinterpretation of their effectiveness and in some instances have been reported as having lost effectiveness (Yildirim et al., 2012; Krattinger et al., 2013). Some time will be spent here in describing these important genes.

*Sr2* was the first APR gene for stem rust to be genetically defined. In the rust atlas (McIntosh et al., 1995) it is described thus.

*“Sr2 is arguably the most important gene for stem rust resistance and one of the most important disease resistance genes to be deployed in modern plant breeding.*”

Such a statement begs examination of the evidence. Before looking into that, some *Sr2* history: starting in 1915, and later partly as a response to disastrous 1919 US stem rust epidemic, McFadden (1930) developed a wheat variety called Hope, released in 1926, derived from a cross between the popular stem rust susceptible North American cultivar Marquis and the highly stem rust resistant tetraploid emmer wheat Yaraslav. Hope was selected for quality and high level stem rust resistance under field conditions. At the time of release, Hope resistance to the then current stem rust strains was considered outstanding and although the variety itself was not successful agronomically, it and a sister line H-44-24 became the source of stem rust resistance in many wheat breeding programs. After Hope resisted the 1935 North American stem rust epidemic, Waldron and Clark (1936) wrote “one need scarcely fear that the reaction to stem rust now found in Hope and so many of its descendants is but a temporary character.” However, although Hope resistance “was considered by many to be permanent and indestructible” (Stakman and Rodenhiser, 1958), the resistance was not magic and the same authors reported that although Hope was highly resistant “during the terrific epidemic of 1935” caused by stem rust race 56, in 1950 race 15B (the Ug99 of the era!) appeared in the US and severely damaged varieties with Hope-derived resistance.

McFadden (1930) did not identify *Sr2* but selected a small combination of genes for stem rust resistance. Later careful glasshouse and field analysis determined the genetic basis of resistance in Hope. These studies identified three R genes *Sr7b*, derived from its Marquis parent and *Sr9b* and a recessive gene *Sr17* derived from the Yaraslav parent (McIntosh et al., 1967; Knott, 1968, 1971). Furthermore, a gene which alone provided partial APR, named *Sr2*, was also identified. Among the segregating lines it was also noted that the level of R gene resistance stage was enhanced (“boosted” to quote Knott) by the presence of *Sr2* and that the high levels of resistance in the field to race 56 resulted from the presence of both the *Sr2* and *Sr9d*. So *Sr2* has two properties, partial APR when used alone and the ability to boost the levels of resistance of R genes. Hope had high levels of resistance to the race 56 epidemic because the R genes were effective against that race. Race 56 overcame the Hope R genes and this host line and its derivatives now expressed only the partial resistance of *Sr2* which was inadequate under stem rust epidemic conditions. *Sr2* was mapped to chromosome 3BS of wheat (Knott, 1971; Hare and McIntosh, 1979). Initially recessive inheritance was reported for *Sr2* but careful studies carried out by Hare and McIntosh (1979) using aneuploids (Chinese Spring lines monosomic and disomic for 3B from Hope) showed that the disomic was more resistant than the monosomic which was in turn more resistant than the Chinese Spring recurrent parent and the inheritance of *Sr2* was described as “partially hemizygous effective,” in other words, the gene had a positive dosage dependent function.

It is this partial (and alone inadequate) resistance component and not the original high levels of resistance in Hope that has proven durable. Nevertheless, Hope and Hope derivatives appear to have had longer lasting effective resistance than their predecessors. The critical question that has never been answered is whether the presence of *Sr2* in the Hope complex makes any contribution to the length of time this complex was durable or alternatively, if its durability (from 1926 to now) was simply the effect of the R gene *Sr9b* in North America and *Sr17* in Australia?

Just as stem rust race 15B overcame Hope resistance in North America in the 1950s due to virulence of this race toward R genes, with the remaining partial resistance of *Sr2* often difficult to see, particularly under high infection pressure, similar observations have been made in recent times in Kenya. *Sr2*, particularly under high spore inoculation pressure, provides very poor levels of resistance to Ug99 when paired with now ineffective R genes like *Sr31* (Pretorius et al., 2000). Nevertheless, the resistance phenotype of *Sr2* is indicated by maintenance of some grain yield and increased 1000 grain weight compared to fully susceptible genotypes during stem rust epidemics (Hare and McIntosh, 1979). *Sr2* alone can also be sufficient for stem rust resistance in places where infection occurs very late in the wheat growing season (McIntosh, personal communication). Although the level of resistance *Sr2* alone is not sufficient under most modern agricultural conditions, it could well make the difference between life and starvation during stem rust epidemics on small farms in the developing world and if used extensively, would contribute to lowering Pgt inoculum levels in the longer term.

*Sr2* entered the CIMMYT wheat breeding program in the late 1940s by way of the North American wheat Newthatch, which combined Hope and Thatcher stem rust resistances. Both Hope and Thatcher have APR (and R genes) derived from tetraploid parents, Yaraslav emmer and Iumillo durum, respectively (Stakman and Rodenhiser, 1958). These sources of APR plus R gene resistance led to varieties that carried *Sr2* and other genes and these CIMMYT varieties remained resistant to stem rust worldwide until the appearance of Ug99 in 1999 and thus *Sr2*, together with other R genes and possibly other poorly defined APR genes, made a major contribution to the green revolution although the exact contribution of *Sr2* to resistance durability is difficult to define.

Durable stem rust resistance has been achieved and continues in North America and Australia, in stem rust prone areas where resistant varieties, many carrying *Sr2*, have been grown for five decades. This was a major breeding achievement in light of the earlier and regular disastrous epidemics. So why did resistance fail so spectacularly in 1999 in Uganda and subsequently in other parts of Africa, parts of the Middle East and Iran? Success it seems was the author of its own failure. Widely used CIMMYT wheat varieties, often represented by closely related genotypes and hence genetically uniform and vulnerable, contained *Sr2* combined with the rye chromosome arm translocation1RS, which provides adaptation and high yield. 1RS also carries the stem rust resistance gene *Sr31*, which was unusual in that in contrast to most R genes, it provided good levels of resistance to all worldwide stem rust strains for over 30 years. In light of this durable resistance combination of *Sr31* and *Sr2* in CIMMYT wheats, continued efforts to increase and diversify sources of stem rust resistance in CIMMYT material had virtually stopped. The prevalence of this single resistance combination in the East Africa and continuous wheat cultivation allowed rapid spread and epidemic development once the virulent Ug99 appeared. To further compound the situation, in addition to overcoming *Sr31*, Ug99 had a very wide spectrum of virulence toward most of the other commonly used R genes and rapidly evolved virulence to the important R genes, *Sr24* and *Sr36*, which has hampered the initial emergency breeding response to incorporate resistance to this strain (Pretorius et al., 2000; Singh et al., 2011b).

Because *Sr2* may effectively boost the resistance phenotypes of R genes (Knott, 1968, 1971), selection for high levels of stem rust resistance in the field results in the stacking of *Sr2* and R genes that are boosted by *Sr2* (and possibly other yet to be defined APR genes) in what some breeders refer to as “the *Sr2* complex.” In fact there were and probably still are (and will be) many different *Sr2* complexes and it was these gene stacks that contributed to varying lengths of resistance durability as long as the R genes in the complex were diverse and provided strong resistance against all current races. If not, the phenotype reverted to *Sr2* partial resistance. Again the fundamental question that has never been answered experimentally is whether the presence of *Sr2* has any direct effect on the durability of the associated stem rust R genes in the complex. The major contribution of *Sr2* may be through the boosting effect on weak race specific R genes that would otherwise be difficult to stack on the basis of their individual phenotypes in the absence of *Sr2*.

Since the occurrence of Ug99, CIMMYT breeders have been working to develop high yielding wheat with high levels of stem rust resistance based on APR and indeed have reported success and about 60% of CIMMYT lines and breeding germplasm carries *Sr2* (Singh et al., 2011a, 2014). The absence of strong and confounding R genes in this breeding approach can be established by selecting against R genes at the seedling stage or by field selection in Kenya with Ug99 derivatives that are virulent to most commonly used R genes. Most of these new lines carry *Sr2* in addition to other quantitative trait locus (QTL) for APR and the hypothesis is that that these new lines display additivity of APR genes. Attempts to explore interactions between *Sr2* and *Lr34* in the background of the wheat genotype, Chinese Spring, revealed increased stem rust severity in derived lines with *Sr2* where the *Lr34* gene has been inactivated, suggesting additive effects between these two APR genes (Lagudah et al., unpublished). There is also a possibility that the new *Sr2* complexes involve weak race specific APR genes, the presence of which may not be obvious without *Sr2*.

So is *Sr2* “*arguably the most important gene for stem rust resistance and one of the most important disease resistance genes to be deployed in modern plant breeding?*” We would tend to agree with this statement on the basis of at least three significant contributions of this gene in complex with R genes. First this gene has been associated with a long period of stem rust free production in the US after the disastrous epidemics of the early 20th century and second its almost 50 years of service to the stem rust free green revolution until the occurrence of Ug99 in East Africa. Third, this gene is already making a major contribution to a new *Sr2* complexes based on stacked APR genes. We are also confident that with current efforts toward identification of diverse additional Sr APR genes highly effective new *Sr2* complexes are achievable by selection for high levels of field resistance during the breeding cycles. The cloning of *Sr2* is being pursued in our lab.

*Lr34* is the most studied APR gene. It was first identified as an APR gene for leaf rust and named by Dyck and Samborski (1979) and mapped to wheat chromosome 7D, although its presence had been recognized in Chinese, Italian, and South American varieties for much longer. Like *Sr2*, *Lr34* enhances (boosts) the effectiveness of R genes for leaf rust resistance (German and Kolmer, 1992; Vanegas et al., 2008). The gene has been cloned and encodes a protein related to the ABC class of trans-membrane transporters (Krattinger et al., 2009). The cargo of the transporter and how it controls disease resistance are currently unknown. *Lr34* was shown to be completely linked to *Yr18* for partial yellow rust resistance and more recently to partial powdery mildew (*Pm38*) and partial stem rust resistance (*Sr57*) and is associated with a leaf tip necrosis phenotype. Point mutations within the *Lr34* gene, developed during its map-based cloning, cause loss of resistance to all these resistances (and leaf tip necrosis), so the one gene provides multiple species resistance that is widely used in wheat cultivars. Thus *Lr34* is the same as the genes designated *Yr18*, *Pm38* and *Sr57,* and *Ltn1* (Krattinger et al., 2009). *Lr34* is therefore a novel class of resistance gene that confers partial resistance to all tested isolates of several pathogen species of wheat and better deserves the description “broad spectrum” resistance than some of the R genes discussed earlier. Perhaps ‘multi pathogen resistance’ even better conveys the nature of these genes. *Sr2*, *Lr46,* and *Lr67* (see below) are also possibly members of this multi-pathogen class.

Having accurate diagnostic markers for *Lr34* has allowed some fascinating “crop forensics” (Krattinger et al., 2011). First, the *Lr34* gene is present in all wheat varieties in several polymorphic full length forms. The form conferring APR differs from the most common susceptibility form (*Lr34-S*) by 2 amino acid changes in two of the 12 trans-membrane domains of the encoded protein. Several mutants were isolated including premature stops and splicing variants that lose *Lr34* resistance so it can be inferred that the resistance form, *Lr34-R* is likely to be a novel gain of function form of the widespread *Lr34*-S allele. The high frequency of *Lr34-R* in Chinese wheat land races relative to other centers of wheat production suggests that the resistance allele arose in China (Kolmer et al., 2008). Its absence in large sample of the wheat D genome donor, *Triticum tauschii*, further suggests that the resistance form arose in hexaploid wheat and not the diploid D genome progenitor. *Lr34* appeared in North American wheat cultivars in the 1960s when the green revolution started.

On the basis of time-tested durability and phenotypic characteristics of just *Lr34* and *Sr2*, some breeders have developed the potentially dangerous view that all APR genes will be durable. On the contrary, it is well documented that some APR genes such as *Lr12*, *Lr13*, *Lr22b*, *and Lr37* are race specific (McIntosh et al., 1995) and others have not been adequately tested. Johnson (1988) also reported that several Yr resistance sources classified as APRs were overcome by new races of stripe rust in Europe and hence were actually race specific. Similar observations of race specificity for different sources of stripe rust APR genes have been reported in North American wheats (Hao et al., 2011; Sthapit et al., 2012) as well as with more recent variation in Pst strains in Europe uncovering race specific APR Yr genes (Sørensen et al., 2014). A newly cataloged gene *Yr49* characterized by our group had all the hallmarks of an APR gene. It provides resistance all Australian stripe rust isolates only at the adult stage but succumbed when tested against stripe rust races in China (Spielmeyer, unpublished). So some APR genes are pathogen isolate (race) specific (or at least environmentally specific) and consequently unlikely to be durable for the same reason single R genes are not durable. Useful levels of APR in crops are probably due to combinations of both non-specific and race specific APR genes. A major challenge in this field is to be able to make accurate predictions as to whether newly identified APR genes will be durable like the long tested *Sr2* and *Lr34* genes.

One of the outstanding, if not surprising outcomes of cloning R genes was that resistance to viruses, bacteria, fungi, oomycetes, nematodes, and sucking insects are nearly all conferred by NB-LRR genes. Several APR genes have been cloned recently from wheat and have broken this uniform mold. Of the three cloned wheat APR genes, all are different and include a cytoplasmic protein kinase gene (*Yr36*, Fu et al., 2009), an ABC transporter gene (*Lr34*, Krattinger et al., 2009) and a non-ABC transporter (*Lr67*, Lagudah, unpublished). All three wheat genes encode functional resistance proteins that can be inactivated by mutation. An important feature of the cloned APR genes is that their phenotypic effects are sufficiently large for fine genetic mapping and isolation of loss of function mutants, both features necessary for map-based cloning (low hanging fruit?). There is a range of weaker APR genes detected only as QTL that do not allow these mapping tools. It will be important to develop genetic stocks and accurate phenotyping tools to map base clone these weaker contributors to APR gene complexes.

Further genes with characteristics similar to *Lr34* have been recently described and accurately mapped. *Lr46* (Singh et al., 1998; Lagudah, 2011) and *Lr67* (Hiebert et al., 2010; Herrera-Foessel et al., 2011, 2014) confer APR against leaf, stripe, and stem rusts, and are completely linked to partial powdery mildew resistance and also show leaf tip necrosis of the flag leaf (*Lr46* is the same as the genes designated *Yr29*, *Sr58* and *Pm39* and similarly, *Lr67* is synonymous with *Yr46*, *Sr55* and *Pm46*). Like *Lr34* these are multi-pathogen APR genes. Another leaf rust APR gene, *Lr68*, has been described but whether it confers multiple disease resistance has not yet been reported (Herrera-Foessel et al., 2012). Given that new APR genes often occur in the same lines as gene complexes of previously described APR genes, having accurate markers for the earlier described genes greatly assists in producing mapping families in which only the new genes segregate. Among these APR genes, *Lr34, Lr46, Lr67,* and *Lr68* confer different levels of partial resistance with *Lr34* generally producing the strongest resistance. It is likely that further important APR components will have weak to very weak phenotypes compared to the current genes so their identification and development of molecular markers becomes more difficult. In these cases, development of stocks in which the weaker genes segregate but stronger genes like *Lr34* are genetically fixed in the background may assist in mapping genes where interactions between *Lr34* and APR genes provide a strong enough phenotype to score above the *Lr34* effect alone.

As mentioned earlier, some genes conferring APR are race specific and not durable. The ability to classify APR as race specific depends on having races that overcome the APR genes and these are often not available. The observation that the durable APR genes *Lr34* and potentially *Sr2*, provide partial resistance to several pathogen species may become a useful trait to distinguish the two classes of APR. So it will be interesting to observe over time this correlation between durability and multi pathogen resistance with the relatively new and untested genes *Lr46* and *Lr67*. Among the 190 or so known examples of rust Sr, Lr and Yr genes, examples of single genes encoding resistance to multiple rust pathogen species are rare (McIntosh et al., 1995). This implies that most Avr gene effectors in the three wheat rust pathogens are diverged. There is only a single known instance, *Sr15* and *Lr20*, where genetics and mutation studies indicate that a single R gene controls race specific resistance to more than one rust species (*Sr15*= *Lr20*, McIntosh, 1977). Therefore multiple pathogen resistance may be a key property that distinguishes the durable class like *Lr34* from the race specific class of APRs.

CIMMYT breeders have championed and successfully practiced breeding for APR to rust diseases for many years and use “the single backcross approach” (Singh et al., 2014). This process, designed to assemble in a single genotype multiple so called “minor” APR genes, has produced breeding lines and varieties with near immunity to rust diseases in the absence of R genes effective against the pathogen races used in the screens (Singh et al., 2014). The wheat lines often also carry genes such as *Sr2, Lr34,* and *Lr46*, combined with unidentified minor genes in adapted high yielding backgrounds. The assembly of “near immunity” levels of resistance to the respective rusts is presumed to depend on the additivity of some of the genes.

Until now the discussion has dealt with R and APR mostly separately but now we need to consider them together in terms of building effective combinations (“stacks” or pyramids”) of both types of genes either independently or together. Some but not all R genes act additively (Roelfs, 1988). For an example of ones that do not, *Sr24* and *Sr26*, both important genes for stem rust control, individually give a type 2 resistance response (pustule formation restricted by surrounding necrosis), and when combined, the response is little different from each gene alone. In a stack of resistance genes with different phenotypes the combined phenotype is commonly determined by the gene with the strongest effect when expressed in a plant as a single gene (Roelfs, 1988). Nevertheless, the use of rust strains avirulent to the first and virulent to the second R gene in a paired combination and *vice versa* shows that both genes function independently (Roelfs, 1988). Whether the genes interact or not, their independent action is the basis of attaining durability of resistance when effective R genes are used in stacks.

It is generally assumed based on the success of breeders in developing strong APR, that APR genes are additive. However, up until recently this assumption has not been stringently tested by experiment with identified genes. We have initiated such studies using accurate DNA markers to make one, two and three gene combinations of *Lr34*, *Lr46,* and *Lr67* in a common background, initially with the aim of determining the best and the minimal combination of these genes for good APR phenotypes. Interestingly the accumulating data for these APR genes are not demonstrating additivity for stripe rust resistance under field conditions. However, we do observe clear additivity between *Lr34* and the race specific APR *Yr49*. There are, however, several reports of clear interactions between APR genes such as *Sr2* or *Lr34* with R genes (Knott, 1968; German and Kolmer, 1992). This leads to the hypothesis that the high levels of APR achieved by CIMMYT and other programs may depend on what we refer to as *foundation* race non-specific APR genes such as *Sr2*, *Lr34*, *Lr46,* and *Lr67*, which boost various (strong and weak) race specific APR genes. The latter genes may include weak NB-LRR genes whose defense signal output even in the presence of corresponding *Avr* genes in the pathogen, are below the threshold of detection on the basis of pathogen growth inhibition. The minor race specific APR genes may not be additive in the absence of the foundation APR genes. However, when stacked with genes like *Lr34*, which probably functions in a different defense pathway to NB-LRR genes, the activity of each R gene could be boosted to a level where visible resistance over and above *Lr34* alone is detectable. Each additional resistance would be race specific. However, if each of these had a different specificity, the gene stack could be durable to pathogens with several corresponding Avr genes.

Another group of genes that could be boosted by race non-specific foundation APRs could include weak autoactive NB-LRR genes discussed earlier. Autoactive genes also provide race non-specific resistance and as discussed above would usually be selected against in breeding programs if their pleiotropic phenotypes such as necrosis or dwarfing were too severe. Nevertheless, such “leaky” R genes with phenotypes below the threshold for detection could also be boosted by non-specific APR genes and accumulated in APR stacks.

A third broad class of naturally occurring genes may contribute to APR effects and could be boosted by foundation APR genes. One arm of the plant immune system is based on pattern recognition receptors that respond to pathogen associated molecular patterns (PAMPs) that are absent in plants, such as the fungal structural molecule, chitin. It is now generally accepted that pathogen evolution toward host adaptation involves expression of effectors that block recognition of signaling steps from pattern recognition receptors (reviewed by Dodds and Rathjen, 2010). It has also been demonstrated that mutations in the plant gene *EDS1* (Falk et al., 1999) increase susceptibility to virulent pathogens. This indicates that pathogens’ effector based suppression of host PAMP induced defense is not complete because an apparent compatible host-pathogen interaction can become more compatible due to host gene mutation. Polymorphisms for “susceptibility” to effector perturbation in host effector targets such as pattern recognition receptors and their downstream defense pathway components that lead to increased levels of defense signals derived from PAMP recognition even in the presence of inhibitory effectors, would be genetically mapped as partial resistance genes. Those polymorphisms associated with increased defense signal transmission could confer varying levels of non-specific resistance and may behave as multi-pathogen APR genes. Whatever the mechanisms, all types of APR genes would be useful in breeding programs. Weak resistance phenotypes may actually be an advantage for the breeding process because this would allow multiple genes of this class to be pyramided on a foundation of ‘strong’ APR genes by selection for the high levels of field resistance achievable not incrementally, but only when a minimum number of genes are combined with known APR genes. The breeding process needs adjustment to increase numbers at early stages so that genotypes with rare combinations of multiple genes occur (Singh et al., 2014). It is important to test these ideas about functions by cloning some of the weak APR genes. One other question is whether *Lr34*, *Lr46*, *Lr67,* and *Sr2* boost the same or different groups of R genes. Interestingly several CIMMYT varieties, such as Parula selected for field resistance (and not with DNA markers), contain all the described “foundation” APR genes *Sr2*, *Lr34*, *Lr46,* and *Lr68*. This could occur because these genes are simply present at high frequencies in CIMMYT breeding germplasm and so have a high probability to occur together in derived lines. Alternatively, these APR genes may have some additive effects under certain conditions so that their combination is selected with high levels of resistance. Another possibility to consider is that each of the strong APR genes may boost different sets of R genes or weak APR genes and so again selection for the highest levels of field resistance would select for the co-presence of the four known APR genes plus unknown weak genes. Will the APR combinations be durable? The answer under the APR-weak R complex hypothesis will depend on the component genes. For example the original gene complex associated with Hope resistance lost maximum activity when race 15B overcame *Sr9d,* which left only the weak residual phenotypic effect of *Sr2*. The important requirement for durability of the new APR complexes will probably depend on the number and diversity of minor genes in and between the gene stacks and prevailing pathogen variation toward R genes in the complex.

## THE FUTURE: BIOTECHNOLOGY AND RUST RESISTANCE BREEDING

Currently selection for resistance to rust in wheat is a major resource consuming activity in most breeding programs and prevents breeders from focusing totally on the critical issue of yield. One current example of biotechnology used to enhance the efficiency of rust resistance breeding is the increasing use of DNA markers to pyramid genes and confirm the presence of genes in, and purity of, released cultivars. This requires DNA markers that are accurate and applicable across wide ranges of, or better still, all of breeders’ germplasm. Many highly accurate makers for wheat rust resistance genes are now available, for example the one used to detect *Sr2* (Mago et al., 2011). There is a very good case for the immediate development of accurate DNA markers for every known and used wheat rust R gene to eliminate the guess work and long timeframes involved in genotype postulation by genetic analysis and multi-pathotype screening. However, phenotypic analysis will still be required in some circumstances where DNA markers alone could be insufficient. For example, we have found lines that express the wild type *Lr34* gene but not rust resistance (Lagudah, unpublished). Given that *Lr34* encodes a transporter, lack of resistance could occur if a second gene, involved in synthesis a hypothesized pathogen inhibitory cargo molecule, were inactive. In a wheat genotype carrying a stack of APR genes including *Lr34*, it would be advantageous to have a simple biochemical marker for *Lr34* function in addition to a DNA marker to test varieties before release. Such non-DNA markers for R genes based on effector expression in plants are discussed below.

Efforts are also being made to develop durable GM solutions to rusts, for example the construction of resistance gene cassettes that incorporate multiple effective genes against each rust species. Such cassettes could include combinations of both R and APR genes and would be delivered to high yielding lines by transformation. One breeding advantage of cassettes is that the genes will segregate as a unit rather than randomly segregating as unlinked stacked genes do during breeding of conventional varieties. The breeding process could involve selection for quality and high levels of yield with fungicide protection and later insertion of cassettes into the genotypes at the end of the process. The breeding process could also involve selection against R genes at the seedling stage then selection for yield and APR in the field and later addition of R gene cassettes at the end of this stage of variety development. Having diverse multiple cassettes will allow one genotype to be rapidly replaced when needed with a near isogenic variety with a different resistance genotype.

Several challenges and requirements are foreseen for cassette breeding such as cloning multiple effective R and APR genes and efficient methods for inserting multiple genes at a single locus. Insertion may either involve constructing multi-gene cassettes *in vitro*, then insertion, or alternatively sequential insertions of several genes at a single target site in the plant genome using genome editing technologies that are currently under development (e.g., Wang et al., 2014). Another advantage of the cassette approach is that it would allow combinations of genes that cannot be selected by breeding, such as genes linked in repulsion in non-recombining regions of the cereal genome (e.g., *Sr31, Sr33, Sr50*). Genes used in this process will come from the richest source, outside of the accessible wheat genome, and include non-host resistance genes. One caution for this approach is, however, provided by experience from classical gene introgressions from diploid to higher ploidy wheat in which the resistance phenotypes of transferred genes are either lower than in the donor species or completely suppressed. For example, a negative interaction between two orthologous NB-LRR-encoding genes that gives rise to suppression of resistance was recently documented at the molecular level. This involves a powdery mildew resistance gene *Pm3* in wheat that suppresses its ortholog *Pm8* transferred into wheat from diploid rye, due to interactions between the encoded proteins (Hurni et al., 2014). Thus, further requirements are rapid procedures for demonstrating component R genes function in the hexaploid wheat, ability to show that genes in the cassette that provide resistance to all common strains of the pathogen genes encode different gene-for-gene specificities (to avoid duplication) and are all functioning in the transgenic lines. For an example of the problem, the alien genes *Sr32* and *Sr39*, both of which are derived from the short arm of chromosome 2 from different accessions of *Aegilops speltoides* and provide resistance against all tested strains of the stem rust pathogen are currently indistinguishable based on visual inspection of infection types and multi-pathotype testing. Are they identical or different specificities? If they are different, they will detect different effectors. Two approaches are possible for distinguishing resistance specificities. The first is by mutation of the pathogen to overcome host resistance. For example a mutant stem rust strain that is virulent to *Sr32* but avirulent to *Sr39* (or *vice versa*) would clearly show that these are different genes. Second, cloning the corresponding Avr genes and developing simple effector delivery systems will be important for these tests (Upadhyaya et al., 2014). For example, a cloned Pgt effector that induced a resistance response when delivered to *Sr32* but not *Sr39* (or *vice versa*) would distinguish these R genes. This type of approach is currently being used in potato breeding to differentiate resistance specificities to late blight conferred by R genes in wild Solanum species (Vleeshouwers et al., 2011) and may also be applicable in wheat to rapidly detect suppressive interactions between transgenes and resident wheat genes similar to that described above for the *Pm3*–*Pm8* interaction.

A further much discussed biotechnology approach, as yet not adequately tested, is host induced gene silencing (HIGS) of essential genes in the pathogen. This approach involves expressing small interfering RNAs in the host that may be transferred to the pathogen and induce silencing of genes important for pathogen virulence. Several publications (see Nunes and Dean, 2012 for review) describe initial attempts to apply this for rust diseases but until the time of writing, all have used transient gene expression systems and demonstration that HIGS will function in transgenic plants is urgently required to provide confidence that this is a viable technology for rust control. Also the exploitation of non-host resistance to wheat rusts that occurs in other grasses, for example rice, is being investigated. At this stage, the nature of the genes involved has not been determined. These approaches are discussed in the accompanying article (Bettgenhaeuser et al., 2014).

There is also the possibility of using of recessive gene resistance that can result from loss or modification of function of host genes that encode protein targets for pathogen effectors and therefore may be essential for virulence. A rust resistance equivalent to the barley mildew resistance gene *Mlo* also comes to mind. No such genes have been identified for wheat and at present almost all wheat rust R genes are dominant. The identification of such recessive genes by mutation of hexaploid wheat would be problematic because of gene triplication, however, mutation screens for rust resistance in barley, a diploid, may reveal host genes necessary for rust pathogen virulence that could then be used in wheat by silencing or mutating the three wheat homoeologs. Recently Wang et al. (2014) demonstrated the feasibility of using gene editing for this purpose by generating a triple mutation of the A, B, and D genome homoeologs of *Mlo* in wheat which conferred strong powdery mildew resistance. Similar approaches targeting triplicated wheat genes identified via the barley mutagenesis approach could be used.

## CONCLUDING REMARKS

The three rust diseases continue to be problems for grain production. Stem rust is the major threat because of the extreme levels of damage the disease causes to susceptible crops and although currently controlled in the world’s major production areas, serious genetic vulnerability exists and active steps are being taken to incorporate new effective resistance in most wheat-growing zones. Stripe rust and leaf rust are not threats but are already serious existing chronic problems and these diseases, especially stripe rust (Hovmoller et al., 2010) are beginning to attract more attention from the international research community. However, in some regions of the developed world there is the view “yield is king” and high yielding rust susceptible varieties are being chosen by farmers with the view that the yield benefit over resistant varieties will more than cover the cost of fungicides in disease years. We are yet to see the scenario of a major epidemic coupled with shortages of rapidly available fungicide supplies for timely chemical control, a situation easily envisioned in developing countries.

There is a continuing discussion about the relative merits of R *versus* APR with simplistic assumptions that all APR genes will be non-race specific, durable and additive in their effect. Certainly the test of time has shown some APR genes like *Sr2* and *Lr34* are durable but their resistance is only partial and mostly insufficient when used alone. Strong resistance achieved by combining these genes with R genes is not durable as shown historically for several gene combinations involving *Sr2*. More recently established effective combinations of partial resistance genes (that may or may not include weak R genes) have been successful in CIMMYT programs but insufficient time has passed since deployment of these new varieties to have experience in their durability. More research is needed to investigate these issues and further develop current and novel approaches for rust resistance. We see the molecular genetic investigation of diverse APR genes, their function and their interactions with weak and strong R genes as a priority area of research for wheat–wheat rust interactions and rust control. The critical questions are what are the “minor” genes present in lines selected in the field for near immunity? Are weak race specific genes included? Do the APR genes we have referred to as ‘foundation APR’ genes like *Lr34* and *Sr2* enhance otherwise undetected R genes and what contribution do the foundation genes make? GM technology and additional genetic analysis will provide the tools for understanding and developing durable rust resistance.

## Conflict of Interest Statement

The authors declare that the research was conducted in the absence of any commercial or financial relationships that could be construed as a potential conflict of interest.
